# Supplementary material for: High-throughput multi-camera array microscope platform for automated 3D behavioral analysis of swimming zebrafish larvae
Source: Commun Biol. 2026 Jan 23;9:141. doi: 10.1038/s42003-025-09421-w (PMC12858796; doi:10.1038/s42003-025-09421-w)
Supplement: Supplementary file 1 — Supplementary Information [file 42003_2025_9421_MOESM1_ESM.pdf]

Supplementary Information

High-throughput multi-camera array microscope platform for automated 3D behavioral analysis of swimming zebrafish larvae

Haitao Chen[1†], Kevin Li[1†], Lucas Kreiss[1], Paul Reamey[2], Lain X. Pierce[3], Ralph Zhang[3], Ricardo Da Luz[3], Amey Chaware[1], Kanghyun Kim[1], Clare B. Cook[1], Xi Yang[1], Joshua F. Lerner[1], Jed Doman[2], Aurélien Bègue[2], John Efromson[2], Mark Harfouche[2], Gregor Horstmeyer[2], Matthew N. McCarroll[3*], and Roarke Horstmeyer[1,2*]

[1]Department of Biomedical Engineering, Duke University, Durham, NC, USA
[2]Ramona Optics Inc., Durham, NC, USA
[3]Department of Pharmaceutical Chemistry, University of California, San Francisco, CA, USA

[†]These authors contributed equally to this work.

[*]Corresponding authors E-mails: matthew.mccarroll@ucsf.edu
roarke.w.horstmeyer@duke.edu

Supplementary Figures

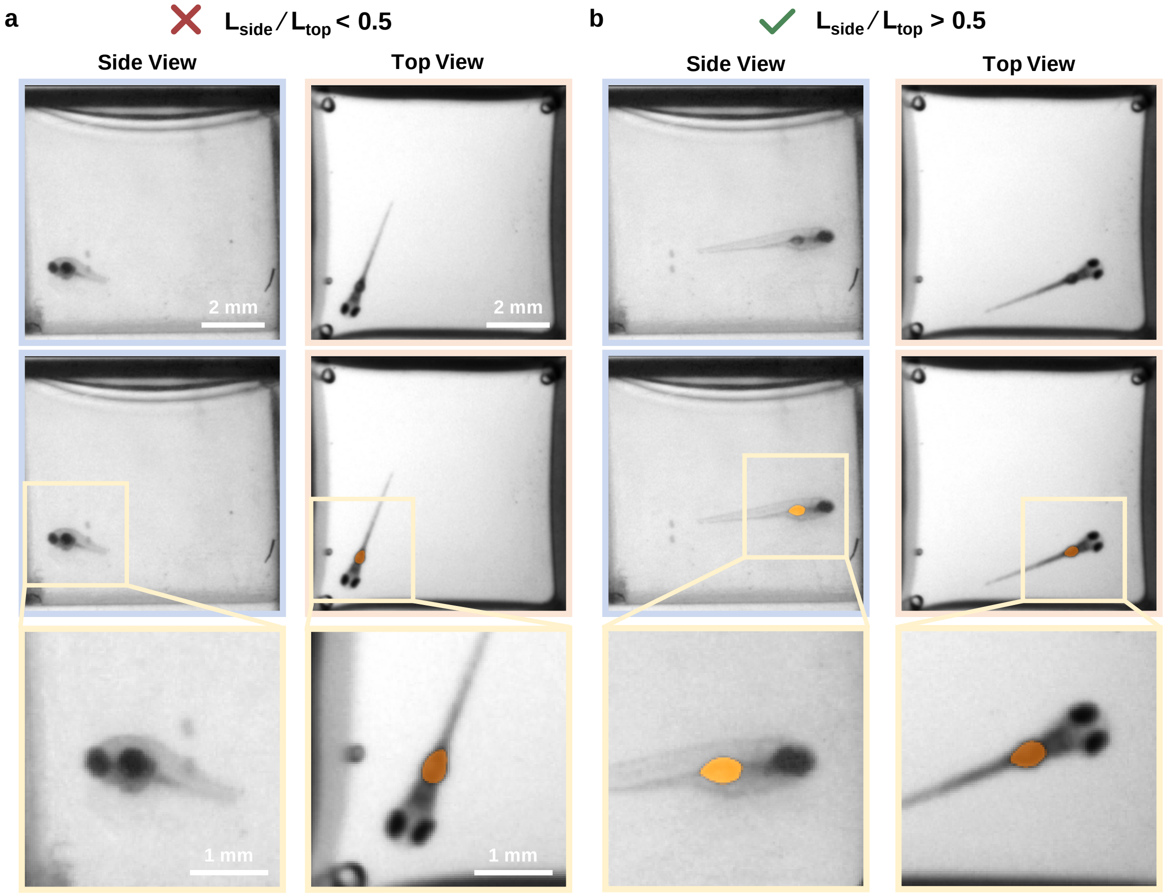

Fig. S1: **Representative examples of our visibility criteria for segmenting the swimbladder. a,** Visibility criteria is not met, the fishes projected length on the side-view is short relative to the true length, resulting in the swimbladder being behind the zebrafish's eyes. **b,** Visibility criteria is met, the long projected lengths in both views ensure that the swim bladder is clearly visible and segmentable.

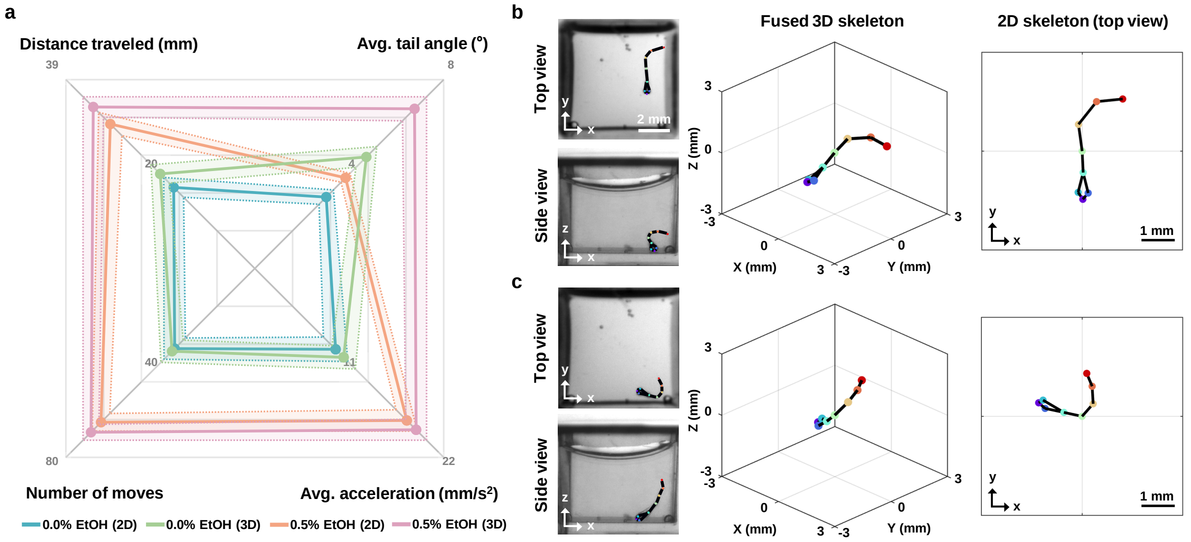

Fig. S2: **Comparison of 2D and 3D kinematic analysis under ethanol exposure using multivariate visualization and skeletal reconstruction. a,** Nightingale plot illustrating the average values and standard errors (SEM, shaded regions) of four key kinematic parameters for zebrafish larvae exposed to 0.0% or 0.5% ethanol (EtOH), measured using 2D and 3D tracking (n = 8 per group). **b-c,** Two examples of larvae exposed to 0.5% EtOH. In **b**, the top view suggests a classic J-turn, while the side view reveals that the movement is actually a C-turn with strong vertical curvature. In **c**, the side view reveals an upward arched tail bend not captured from above.
