# Supplementary material for: High-throughput multi-camera array microscope platform for automated 3D behavioral analysis of swimming zebrafish larvae
Source: Commun Biol. 2026 Jan 23;9:141. doi: 10.1038/s42003-025-09421-w (PMC12858796; doi:10.1038/s42003-025-09421-w)
Supplement: Supplementary file 2 — Description of Additional Supplementary Files [file 42003_2025_9421_MOESM2_ESM.pdf]

Description of Additional Supplementary File

File name: Supplementary Data 1
Description: The source data behind the graphs in the paper

File name: Video 1
Description: System principles, 3D skeletal tracking, and swim bladder morphodynamics
