# Supplementary material for: High-throughput multi-camera array microscope platform for automated 3D behavioral analysis of swimming zebrafish larvae
Source: Commun Biol. 2026 Jan 23;9:141. doi: 10.1038/s42003-025-09421-w (PMC12858796; doi:10.1038/s42003-025-09421-w)
Supplement: Supplementary file 5 — Reporting Summary [file 42003_2025_9421_MOESM5_ESM.pdf]

nature portfolio

Corresponding author(s): Matthew N. McCarroll, Roarke Horstmeyer

Last updated by author(s): Dec 3, 2025

Reporting Summary

Nature Portfolio wishes to improve the reproducibility of the work that we publish. This form provides structure for consistency and transparency in reporting. For further information on Nature Portfolio policies, see our Editorial Policies and the Editorial Policy Checklist.

Statistics

For all statistical analyses, confirm that the following items are present in the figure legend, table legend, main text, or Methods section.

n/a	Confirmed	
☐	☒	The exact sample size (*n*) for each experimental group/condition, given as a discrete number and unit of measurement
☐	☒	A statement on whether measurements were taken from distinct samples or whether the same sample was measured repeatedly
☐	☒	The statistical test(s) used AND whether they are one- or two-sided<br>*Only common tests should be described solely by name; describe more complex techniques in the Methods section.*
☒	☐	A description of all covariates tested
☒	☐	A description of any assumptions or corrections, such as tests of normality and adjustment for multiple comparisons
☐	☒	A full description of the statistical parameters including central tendency (e.g. means) or other basic estimates (e.g. regression coefficient) AND variation (e.g. standard deviation) or associated estimates of uncertainty (e.g. confidence intervals)
☐	☒	For null hypothesis testing, the test statistic (e.g. *F*, *t*, *r*) with confidence intervals, effect sizes, degrees of freedom and *P* value noted<br>*Give P values as exact values whenever suitable.*
☒	☐	For Bayesian analysis, information on the choice of priors and Markov chain Monte Carlo settings
☒	☐	For hierarchical and complex designs, identification of the appropriate level for tests and full reporting of outcomes
☒	☐	Estimates of effect sizes (e.g. Cohen's *d*, Pearson's *r*), indicating how they were calculated

Our web collection on statistics for biologists contains articles on many of the points above.

Software and code

Policy information about availability of computer code

Data collection	Ramona Optics MCAM Software
Data analysis	Skeletal tracking: https://github.com/DeepLabCut/DeepLabCut<br>Swim bladder segmentation: https://github.com/facebookresearch/sam2<br>Behavioral kinematics: MATLAB R2024b

For manuscripts utilizing custom algorithms or software that are central to the research but not yet described in published literature, software must be made available to editors and reviewers. We strongly encourage code deposition in a community repository (e.g. GitHub). See the Nature Portfolio guidelines for submitting code & software for further information.

Data

Policy information about availability of data

All manuscripts must include a data availability statement. This statement should provide the following information, where applicable:

- Accession codes, unique identifiers, or web links for publicly available datasets
- A description of any restrictions on data availability
- For clinical datasets or third party data, please ensure that the statement adheres to our policy

Source data underlying graphs can be obtained from Supplementary Data 1. All other data are available from the corresponding authors on reasonable request.

Research involving human participants, their data, or biological material

Policy information about studies with human participants or human data. See also policy information about sex, gender (identity/presentation), and sexual orientation and race, ethnicity and racism.

Reporting on sex and gender	N/A
Reporting on race, ethnicity, or other socially relevant groupings	N/A
Population characteristics	N/A
Recruitment	N/A
Ethics oversight	N/A

Note that full information on the approval of the study protocol must also be provided in the manuscript.

Field-specific reporting

Please select the one below that is the best fit for your research. If you are not sure, read the appropriate sections before making your selection.

☒ Life sciences ☐ Behavioural & social sciences ☐ Ecological, evolutionary & environmental sciences

For a reference copy of the document with all sections, see nature.com/documents/nr-reporting-summary-flat.pdf

Life sciences study design

All studies must disclose on these points even when the disclosure is negative.

Sample size	Sample sizes (n = 8 per group) were chosen based on prior studies with similar behavioral variability in zebrafish larvae, and were sufficient to detect drug-induced behavioral changes under our imaging conditions.
Data exclusions	All larvae were included unless there was a clear imaging artifact or failure in tracking or segmentation, both of which were well-defined in the analysis pipeline.
Replication	Reproducibility was accomplished by having multiple researchers handle the behavioral recording paradigm. Similar behavioral trends and drug responses were observed across replicates. Everything was reproducible and robust.
Randomization	Larvae were randomly assigned to treatment or control groups before imaging. Wells were distributed to minimize positional bias across the plate.
Blinding	Data analysis was performed using an automated pipeline without manual intervention. Group labels were blinded during initial data processing and tracking validation.

Reporting for specific materials, systems and methods

We require information from authors about some types of materials, experimental systems and methods used in many studies. Here, indicate whether each material, system or method listed is relevant to your study. If you are not sure if a list item applies to your research, read the appropriate section before selecting a response.

Materials & experimental systems

n/a	Involved in the study	
☒	☐	Antibodies
☒	☐	Eukaryotic cell lines
☒	☐	Palaeontology and archaeology
☐	☒	Animals and other organisms
☒	☐	Clinical data
☒	☐	Dual use research of concern
☒	☐	Plants

Methods

n/a	Involved in the study	
☒	☐	ChIP-seq
☒	☐	Flow cytometry
☒	☐	MRI-based neuroimaging

Animals and other research organisms

Policy information about studies involving animals; ARRIVE guidelines recommended for reporting animal research, and Sex and Gender in Research

Laboratory animals	All zebrafish larvae (Danio rerio) were of the wild-type Singapore strain and were tested at 7 days post-fertilization.
Wild animals	N/A
Reporting on sex	Sex of larvae was not determined due to early developmental stage (7 dpf)
Field-collected samples	N/A
Ethics oversight	All zebrafish procedures were performed in accordance with protocols approved by the University of California, San Francisco (UCSF) Institutional Animal Care and Use Committee (IACUC), and in compliance with the Guide for the Care and Use of Laboratory Animals (National Institutes of Health, 1996). We have complied with all relevant ethical regulations for animal use.

Note that full information on the approval of the study protocol must also be provided in the manuscript.

Plants

Seed stocks	N/A
Novel plant genotypes	N/A
Authentication	N/A
